# Expression Patterns and Clinical Relevance of HSP70 and Metallothionein in Triple-Negative and Luminal A Breast Cancer: A Croatian Cohort Study

**DOI:** 10.3390/cells15040351

**Published:** 2026-02-15

**Authors:** Sara Bilić Knežević, Tamara Gulić, Damir Grebić, Mirisa Tokić, Manuela Avirović, Anita Savić-Vuković, Marin Marinović, Davor Jurišić, Dalibor Broznić

**Affiliations:** 1 Department of Oncology and Radiotherapy, Dubrava University Hospital, Avenija Gojka Šuška 6, 10000 Zagreb, Croatia; sbknezevic@kbd.hr; 2 Department of Physiology and Immunology, Faculty of Medicine, University of Rijeka, Braće Branchetta 20, 51000 Rijeka, Croatia; tamara.gulic@uniri.hr; 3 Department of General and Oncological Surgery, Clinical Hospital Center Rijeka, Krešimirova 42, 51000 Rijeka, Croatia; damir.grebic@uniri.hr; 4 Department of Surgery, Faculty of Medicine, University of Rijeka, Braće Branchetta 20, 51000 Rijeka, Croatia; 5 Department of Oncology and Nuclear Medicine, General Hospital Zadar, Bože Peričića 5, 23000 Zadar, Croatia; mirisa.tokic@bolnica-zadar.hr; 6 Department of General Pathology and Pathologic Anatomy, Faculty of Medicine, University of Rijeka, Braće Branchetta 20, 51000 Rijeka, Croatia; manuela.avirovic@uniri.hr (M.A.); anitasv@uniri.hr (A.S.-V.); 7 Department of Traumatology, Faculty of Medicine, University of Rijeka, Braće Branchetta 20, 51000 Rijeka, Croatia; marin.marinovic1@uniri.hr; 8 Department of Plastic and Reconstructive Surgery, Clinical Hospital Center Rijeka, Krešimirova 42, 51000 Rijeka, Croatia; dr.davor.jurisic@gmail.com; 9 Department of Medical Chemistry, Biochemistry and Clinical Chemistry, Faculty of Medicine, University of Rijeka, Braće Branchetta 20, 51000 Rijeka, Croatia; 10 Department of Environmental Health, Teaching Institute of Public Health of Primorje-Gorski Kotar County, Krešimirova 52a, 51000 Rijeka, Croatia

**Keywords:** metallothionein, HSP70, breast cancer, triple-negative breast cancer, Luminal A subtype, cellular stress response, tumor biology

## Abstract

**Highlights:**

**What are the main findings?**

- Nuclear expression of HSP70 is significantly higher in malignant breast cancer specimens than in benign lesions, regardless of molecular subtype.
- Cytoplasmic expression of metallothionein is significantly lower in TNBC and Luminal A carcinomas, without independent prognostic value.

**What are the implications of the main findings?**

- HSP70 and metallothionein expression patterns reflect cellular adaptation to stress rather than serving as independent prognostic biomarkers in breast cancer.
- The results emphasize the importance of protein subcellular localization and an integrative approach in breast cancer biomarker research.

**Abstract:**

Metallothioneins (MTs) and heat shock protein 70 (HSP70) are key regulators of cellular stress response and metal homeostasis and play important roles in tumor biology. The aim of this study was to examine their expression patterns and potential prognostic significance in different molecular subtypes of breast cancer (BC), with special emphasis on triple-negative breast cancer (TNBC) and the Luminal A subtype, compared with benign breast lesions (fibroadenomas). A total of 90 tissue samples were included, and the expression of MTs in the cytoplasm and nucleus and HSP70 in the nucleus of tumor cells was analyzed immunohistochemically and correlated with clinicopathological features and treatment outcomes. Distinct expression patterns of HSP70 and MTs were observed between malignant and benign samples, as well as among the analyzed molecular subtypes of BC, suggesting their involvement in cellular adaptive mechanisms associated with malignant transformation. TNBC was characterized by less favorable clinicopathological features compared to the Luminal A subtype, including higher histological grade, increased proliferative activity, and a higher incidence of recurrence and metastatic disease. Survival analyses confirmed a worse outcome for patients with TNBC, while HSP70 and MTs expression did not show independent prognostic value in multivariate models. In conclusion, although HSP70 and MTs play important biological roles in the cellular response to stress and tumor adaptation, their expression in this study does not represent an independent prognostic indicator of clinical outcome. Nevertheless, the observed expression patterns provide insight into the complex mechanisms of tumor adaptation and emphasize the need for integrative approaches in BC biomarker research.

## 1. Introduction

Breast cancer (BC) is the most commonly diagnosed malignancy among women worldwide and remains a leading cause of cancer-related mortality, with more than 2.3 million newly diagnosed cases and approximately 670,000 deaths annually [1,2,3]. In Europe, BC incidence is among the highest globally, with considerable regional variation, while mortality rates have declined in Western Europe but remain relatively high in Eastern Europe, largely reflecting differences in early detection and access to therapy [2,4,5,6]. In Croatia and the surrounding region, approximately 2700 new BC cases are diagnosed each year, with an incidence rate of 85–90 per 100,000 women and mortality rates remain among the highest in the EU despite recent improvements in screening and therapeutic approaches [7,8].

Prat et al. proposed a classification of five intrinsic molecular BC subtypes–Luminal A, Luminal B, HER2-enriched, Basal-like, and Claudin-low–based on gene expression profiles, highlighting the high molecular and biological heterogeneity of this disease [9,10]. These subtypes differ in the expression of estrogen receptor (ER), progesterone receptor (PR), and human epidermal growth factor receptor 2 (HER2), as well as levels of proliferative index (Ki-67) and clinical outcomes [9,11,12]. This classification is currently used in clinical practice, improving predictions of disease progression and response to therapy [9,11,12,13]. Additionally, Ellsworth et al. found substantial genomic variations across primary tumors, metastatic tissues, and circulating tumor cells, which affect resistance to therapy [14]. This molecular heterogeneity has important implications for clinical practice, as multiple triple-negative BC (TNBC) subtypes with specific mutation patterns, characteristic signaling pathways, and variable prognoses have been identified [12,13,14,15].

Luminal A cancers comprise approximately 40–60% of all diagnosed BCs, making this subtype one of the most frequent molecular categories [9,10,16]. They are usually ER and PR positive, do not overexpress HER2, and have a low Ki-67 index, which is associated with a five-year survival probability of more than 80% [11,12]. However, in Croatia, robust national-level analyses of the prevalence, characteristics, and survival outcomes of patients diagnosed with the Luminal A subtype are still largely lacking. In contrast, TNBC accounts for about 10–20% of BCs and is typically characterized by a lack of ER, PR, and HER2 expression [10,15]. For patients with metastatic TNBC, the median overall survival ranges from 13 to 18 months, which is markedly shorter than that observed in other molecular subtypes of BC [17,18,19]. Nevertheless, despite these adverse biological features, a retrospective Croatian cohort study by Tečić Vuger et al. reported that TNBC represents 16% of all BC cases, with a five-year survival rate of 74% (95% CI 66–81%) [20,21].

Current research on molecular markers in BC is increasingly focusing on proteins that help cells manage stress and maintain homeostasis, as their dysregulation can drive more aggressive tumor behavior [22,23,24,25]. Among these, metallothioneins (MTs) and heat shock proteins (HSPs) are emerging as important candidates for predicting prognosis and treatment response, particularly in TNBC.

Metallothioneins are small, cysteine-rich proteins encoded on chromosome 16q13, with four main isoforms (MT1–MT4) that differ in tissue distribution and function [26]. They help cells cope with oxidative stress, detoxify heavy metals, and maintain metal ion balance, while their expression can be triggered by metals, glucocorticoids, cytokines, and oxidative stimuli [26,27]. In addition to their protective role against stress damage, MTs are involved in growth, differentiation, apoptosis, and angiogenesis–processes frequently dysregulated in cancer [26,28]. Overexpression of MTs has been linked to high tumor grade, rapid proliferation, chemoresistance, and poor prognosis [26,27]. Specifically, in TNBC, overexpression of MT1/2 and MT3 is associated with more aggressive tumors, higher histological grade, and worse overall survival [28,29]. Interestingly, MT3 overexpression may also reduce invasiveness by regulating matrix metalloproteinases [30,31].

Heat shock protein 70 (HSP70) similarly supports tumor progression, metastasis, and therapy resistance by stabilizing oncoproteins, blocking apoptosis, and helping cancer cells survive under stressful conditions [23,32]. Elevated HSP70 expression correlates with worse prognosis and shorter disease-free survival and is therefore frequently investigated as a potential therapeutic target [22,23]. In addition to its intracellular functions, HSP70 influences the tumor immune microenvironment by promoting immunosuppressive cell populations and protumorigenic functions of macrophages, particularly following chemotherapy exposure [33,34]. In TNBC, HSP70 has been shown to contribute to increased tumor cell plasticity and adaptive capacity, thereby facilitating therapeutic resistance and disease progression [35]. Furthermore, overexpression of HSP70 has been associated with the induction of forkhead box P3 (FOXP3+) immunosuppressive T cells and increased programmed death-ligand (PD-L1) expression in the tumor microenvironment [36], while chemotherapy-induced HSP70 secretion may promote protumorigenic functions of macrophages and serve as a potential predictor of disease recurrence [33,34].

Previous studies have primarily examined the individual roles of MTs and HSP70 proteins in BC progression. However, data on the joint prognostic potential of MTs and HSP70 proteins, as well as their interaction with different molecular subtypes of BC, remain very scarce. A meta-analysis by Dimas et al. indicates that most existing studies have mainly focused on the individual roles of different stress proteins [22]. Furthermore, Kabakov et al. emphasize that, although the functions of HSP70 are well known, studies analyzing the synergism between HSP70 and MT and their impact on different subtypes of BC are lacking [23]. Peterko et al. examined HSP70 expression in TNBC and benign breast lesions but did not simultaneously assess MT expression [37], highlighting how little is currently known about possible synergistic interactions between these two stress-response proteins.

So far, HSP70 and MTs have mostly been investigated separately in TNBC, and their combined, potentially synergistic prognostic value has not yet been systematically explored. Addressing this gap, the present study aimed to assess HSP70 and MTs expression in TNBC and Luminal A BCs and to compare these findings with benign breast lesions, including fibroadenomas. In addition, the study aimed to explore how the expression of these proteins relates to key clinicopathological parameters, the development of metastases and recurrences, and survival outcomes, in order to better clarify their biological role and prognostic relevance in BC.

## 2. Materials and Methods

### 2.1. Study Design and Ethical Approval

The study was conducted in accordance with the core principles of bioethics–autonomy, beneficence, non-maleficence, and justice–as well as additional principles such as confidentiality and respect for the dignity and integrity of participants. It met all ethical requirements outlined in the Nuremberg Code and the latest version of the Declaration of Helsinki governing research involving human subjects. Medical and personal data were handled according to current bioethical standards, with particular attention to anonymizing records and safeguarding the confidentiality of all participant information. Tissue samples were used exclusively for scientific purposes and had been previously processed and archived in accordance with relevant institutional procedures. Ethics approval was obtained from the Ethics Committee of the Clinical Hospital Center Rijeka, Croatia (approval number: Klasa: 003-05/21-1/39; Ur.broj: 2170-29-02/1-21-2; approval date: 17 March 2021) and the Committee for Ethical Issues of the Faculty of Medicine, University of Rijeka, Croatia (approval number: Klasa: 003-08/21-1/27; Ur.broj: 2170-24-04-3-21-4; approval date: 27 April 2021).

### 2.2. Study Population

This retrospective study included patients treated for BC at the Clinical Hospital Center Rijeka (Croatia) from 2011 to 2019. The diagnosis of BC was confirmed by histopathological examination, and immunophenotypic characterization of the tumors was performed according to established diagnostic standards.

The study included 60 patients: 30 diagnosed with Luminal A BC and 30 with TNBC. For comparison, a control group (fibroadenomas–FA) consisted of 30 non-cancerous breast tissue samples. These control samples were obtained from diagnostic biopsies performed due to mammographically detected microcalcifications, which were histologically confirmed as non-proliferative lesions such as microcysts, cysts, fibrosis, and fibrocystic changes.

Age and menopausal status at the time of study inclusion, as well as baseline demographic and clinical characteristics for each patient, were documented. A detailed pathology review included: histopathological classification of the cancer (ductal carcinoma, carcinoma of no special type, medullary carcinoma, metaplastic carcinoma), nuclear grade, ER and PR status, and HER2 status. In addition, the Ki-67 index, presence of lymphovascular invasion, and perineural invasion were assessed. Furthermore, information on surgical and adjuvant therapy (chemotherapy, hormonotherapy, radiation therapy) was collected from patient medical records. Data on the timing and pattern of metastatic progression and disease recurrence were also recorded.

### 2.3. Inclusion and Exclusion Criteria

The study included female patients diagnosed with either Luminal A or TNBC, aged between 30 and 70 years. These patients had primary tumors classified as T2 or T3 with lymph node involvement categorized as N0–N3 according to the TNM (Tumor Node Metastasis) classification system, and no distant metastases at diagnosis. T2 tumors measure 2 to 5 cm, while T3 tumors are larger than 5 cm. The N classification for regional lymph node involvement is as follows: N0 indicates no nodes involved; N1 indicates 1 to 3 axillary nodes or microscopic internal mammary nodes; N2 involves 4 to 9 axillary nodes or clinically detectable internal mammary nodes; and N3 involves 10 or more axillary nodes or other regional nodes such as infraclavicular, supraclavicular, or a combination of these.

Patient characteristics recorded included age, menopausal status, tumor histology (ductal, “no special type” [NST], medullary, metaplastic), nuclear grade, hormone receptor status (ER, PR), HER2 status, Ki-67 proliferation index, and evidence of lymphovascular and perineural invasion. Clinical data also included information on surgical procedures (date and type of surgery) and adjuvant therapies administered (chemotherapy, hormonal therapy, radiotherapy), as well as the timing and sites of metastatic progression and disease recurrence.

Exclusion criteria were inadequately preserved biopsy samples, other histological carcinoma types (e.g., invasive lobular carcinoma, Luminal B), non-invasive tumors (in situ), tumors at stages T1 or T4, patients with distant metastases at diagnosis, those who had undergone preoperative systemic antineoplastic therapy, patients with a history of malignant disease, and patients with recurrent disease.

### 2.4. Tissue Sampling and Preparation

The BC tissue samples were embedded according to the standard protocol of the Clinical Institute of General Pathology and Pathological Anatomy, Faculty of Medicine, University of Rijeka. Tissue microarrays (TMA) were prepared from the examined material, with each tumor biopsy represented by two cylinders of primary BC tissue, each 1.5 mm in diameter. Benign breast changes were represented by one cylinder per case. Subsequently, Formalin-Fixed Paraffin-Embedded (FFPE) TMA blocks were serially sectioned into 4 µm thick slices using a sliding microtome (Leica SM 2010 R, Leica Biosystems, Nussloch, Germany) and transferred onto silanized glass slides. During the immunohistochemical process, some cores were lost or fragmented, so the number of examined samples sometimes varied between variables.

### 2.5. Immunohistochemical and Fluorescence Labeling

Immunohistochemical staining was performed on tumor tissues to identify MTs and HSP70 in the cytoplasm and nuclei of cells. The antibodies and ligands used included human IgG1 anti-methionine (MT I + II) monoclonal antibody (mAb), mouse IgG1 anti-actin mAb, rabbit IgG1 anti-tubulin mAb (all from Abcam, Cambridge, UK) and rhHSP70 proteins (Sigma-Aldrich, St. Louis, MO, USA). Actin, serving as a cytoskeletal marker, was labeled alongside tubulin in HSP70-influenced cell preparations.

After binding of the primary antibodies, the cell samples were labeled with secondary anti-mouse and anti-rabbit antibodies conjugated to the appropriate fluorochrome, either green fluorescence using Alexa Fluor 488 or red fluorescence using Alexa Fluor 594 (Thermo Fisher Scientific, Waltham, MA, USA). Cell nuclei were visualized with 4′,6-diamidino-2-phenylindole (DAPI; Sigma-Aldrich, St. Louis, MO, USA), which exhibits blue fluorescence, and samples were embedded in Mowiol medium (Hoechst, Odenwald, Germany).

Observations were conducted using an Olympus BX51 fluorescence microscope (Olympus, Tokyo, Japan). Immunohistochemical labeling of MT antigens was examined with a light microscope (Olympus DP50, Tokyo, Japan) using Cell Imaging software, version 3.0 (Olympus, Tokyo, Japan). Microphotographs representing the findings were captured at 100× and 400× magnifications to visualize the pattern of tissue and cellular marker labeling.

### 2.6. Quantification and Scoring of Protein Expression

Positive cells for each marker within the tissue were identified semi-quantitatively using the integrated Alphelys Spot Browser 2 system (Alphelys, Plaisir, France). For each sample, results were reported as the proportion of positively stained cells relative to the total number of cells examined. Staining intensity in the cytoplasm and nucleus was graded on a three-point scale (1–3). To minimize variability, two pathologists independently assessed all slides, blinded to clinical and other study data. Any discrepancies between their evaluations were reviewed and resolved by mutual agreement.

### 2.7. Statistical Analysis

Statistical analysis was performed using the Statistica^®^ software package, Version 14.0 (TIBCO Software Inc., Palo Alto, CA, USA). Categorical variables are presented as absolute frequencies, while continuous variables are reported as median values with interquartile ranges due to their non-normal distribution. The Mann–Whitney U test was used to compare two independent groups, and the Kruskal–Wallis test was used for comparisons involving more than two groups. Non-parametric correlation analysis between selected variables was conducted using the Kendall Tau test.

The overall survival (OS) and disease-free survival (DFS) were analyzed by the Kaplan–Meier method, with cumulative survival plots compared between patients with TNBC and Luminal A BC. Differences in survival curves were tested using the log-rank test.

Univariate Cox regression analysis was performed to assess the association of clinicopathological variables and biomarkers with DFS. Variables included in the multivariate Cox model were selected based on the results of the univariate analysis and clinical relevance, with stratification by histological grade due to violation of the proportional hazards assumption. Variables included in the multivariate Cox regression model were selected based on borderline or statistically significant associations in the univariate analysis (*p* < 0.10) and clinical relevance. Due to the limited number of events and potential collinearity among biologically related variables such as histological grade, Ki-67 index, and molecular subtype, these parameters were not included simultaneously in the same model to avoid model instability.

All statistical analyses were two-sided, and statistical significance was set at *p* < 0.05.

## 3. Results

### 3.1. Comparative Analysis of Carcinoma Characteristics, Recurrence, Metastasis, and Survival Outcomes in Triple-Negative (TNBC) and Luminal A Breast Cancers

Table 1 presents the demographic and clinicopathological characteristics of three study groups (*n* = 30 each): TNBC and Luminal A BC subtypes, and fibroadenomas (FA) serving as the control group. To identify clinicopathological differences among the groups, age distribution, tumor size, lymph node status, histological grade, and the Ki-67 proliferation index were evaluated.

Histopathological analysis showed that the most common cancer was invasive ductal carcinoma, found in 43 patients. Non-special type (NST) carcinoma was diagnosed in 10 patients, while medullary and metaplastic carcinomas were observed in 3 and 4 patients, respectively. In the TNBC group, 23 patients had the ductal histotype, while the remaining patients had either metaplastic or medullary cancers. Within the Luminal A subgroup, most patients had ductal histology, and the others were diagnosed with NST.

Demographic analysis showed an equal distribution of patients across different age groups. The youngest patients were 18 years old, and the oldest were 70. The largest group consisted of patients aged 61 to 70 years, comprising 30 patients, followed by those aged 51 to 60 years, with 22 patients. There were very few patients under 30 years old, with only 13 cases. Considering age distribution by carcinoma type, in the TNBC group, most patients (14) were aged 61 to 70, while in the Luminal A group, the majority (16) were in the same age range. The control group with FA was predominantly composed of younger patients, most of whom were between 20 and 40 years old.

Clinical and histopathological characteristics, lymph node involvement, and Ki-67 proliferative index in TNBC and Luminal A BC subtypes are shown in Table 2. Most patients had tumors between 2 and 5 cm in size, corresponding to the pT2 stage, observed in 54 cases. In contrast, a minority of 6 patients had tumors larger than 5 cm (pT3). Examining cancer size by subtype, among TNBC patients, 28 had pT2 tumors and 2 had pT3 tumors. Among Luminal A patients, 26 had pT2 tumors and 4 had pT3 tumors. The pathological status of the lymph nodes was also assessed. Among TNBC patients, 20 had negative lymph node status (pN0), 5 had pN1 status (1–3 involved lymph nodes), 2 were classified as pN2 (4–6 affected lymph nodes), and 2 as pN3 (>7 affected lymph nodes). Lymph node status was unknown for one patient (pNx). Among Luminal A patients, 11 had no lymph node involvement (pN0), 11 were pN1, 3 were pN2, 3 were pN3, and 2 had undetermined status. Regarding histological grades, the TNBC group included 14 G2 and 16 G3 lesions, indicating a more aggressive disease. Conversely, the Luminal A group was predominantly lower grade: 18 G1, 8 G2, and 4 G3.

The Ki-67 proliferation index, an important parameter for determining biological aggressiveness, was high (>20%) in 25 TNBC patients and low (<20%) in 5. In contrast, all Luminal A patients had a Ki-67 index below 20%, consistent with the less aggressive biological behavior of these tumors. A cut-off value of 20% was applied in accordance with commonly used thresholds in clinical practice and previous studies distinguishing low- and high-proliferative breast cancers [38,39,40,41].

Table 3 presents disease outcomes, including recurrence, metastasis, disease-free survival (DFS), and overall survival (OS) in TNBC and Luminal A BC subtypes. Recurrence and metastasis data show that TNBC patients had higher rates of both compared with the Luminal A group. Nine patients in the TNBC group had metastases or recurrences, while the remaining 21 patients were disease-free. In the Luminal A subgroup, 26 patients were disease-free, while 4 experienced metastases or recurrence. Among metastasis locations, combined metastases were most prevalent in the TNBC group (6 patients), with bone, distant lymph nodes, and the CNS as other sites. Metastases were less frequent in the Luminal A group, occurring mainly in the liver and bone. OS and DFS were assessed over 60 months. Eleven of the 30 TNBC patients did not achieve DFS at 60 months, while 19 remained disease-free. In the Luminal A group, three patients had a relapse, and 27 remained disease-free during the follow-up period. Regarding OS, in the TNBC group, 10 patients died and 20 survived for 60 months, whereas in the Luminal A group, one patient died and 29 survived throughout the follow-up period.

### 3.2. Comparative Analysis of HSP70 and Metallothionein Protein Expression in Triple-Negative (TNBC), Luminal A, and Fibroadenomas Samples

**Figure 1:**
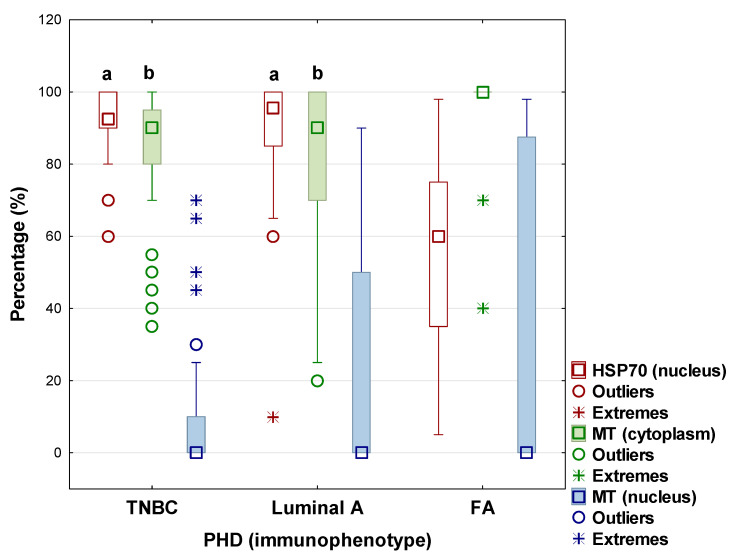
Distribution of heat shock protein 70 (HSP 70) and metalothionein (MT) expression in nucleus and cytoplasm by median, interquartile range (25–75%), and whiskers representing non-outlier range across triple-negative breast cancer (TNBC), Luminal A carcinoma, and fibroadenoma (FA) subtypes, with statistical significance denoted by a and b (*p* < 0.05) relative to control group (FA).

As an initial analysis, the expression of several proteins (HSP70 in the nucleus and MTs in the nucleus and cytoplasm) was compared across three groups of breast tissue samples: TNBC, Luminal A carcinoma, and a control group with fibroadenoma (FA). The aim was to identify differences in protein expression between the control group and each cancer type, as well as among the cancer types themselves. The results are shown in Figure 1. Because the data were not normally distributed, the Kruskal–Wallis test was used for group comparisons, followed by post hoc tests.

The findings showed that the nuclei of both cancer groups (Luminal A and TNBC) had significantly higher levels of HSP70 protein expression than the control group (H = 38.16; *p* < 0.001). The mean ranks for Luminal A and TNBC were 58.45 and 58.26, respectively, while the control group (FA) had a lower mean rank of 20.56. Pairwise post hoc comparisons confirmed statistically significant differences between all groups (*p* < 0.05), allowing a clear distinction between malignant and benign lesions. In contrast, there was no significant difference between TNBC and Luminal A, indicating that nuclear HSP70 protein overexpression is associated with malignancy in general rather than a specific BC subtype.

Analysis of cytoplasmic MT protein expression also revealed statistically significant group differences (H = 24.14; *p* < 0.001). The highest mean rank was recorded for the control group (FA) at 68.04, while TNBC and Luminal A carcinoma patients had much lower values (37.85 and 40.56, respectively). Post hoc tests confirmed significant differences among all groups, indicating that cytoplasmic MT protein expression significantly decreases in malignant lesions compared to benign fibroadenomas. Similar to nuclear HSP70, no significant difference was found between the TNBC and Luminal A subtypes.

Furthermore, no statistically significant difference was found among the groups for MT protein expression in the nucleus (H = 3.81; *p* = 0.1487). The mean ranks for TNBC, Luminal A, and FA were 40.96, 50.18, and 52.13, respectively, indicating that nuclear MT protein expression does not correlate with any specific patient group.

### 3.3. Correlation of Histological and Clinical Parameters with Disease Outcomes in TNBC and Luminal A Breast Cancer: A Kendall Tau Analysis

Correlations between clinical and biological characteristics in two BC subtypes, TNBC and Luminal A, were examined using Kendall Tau correlation analysis. This analysis assessed the associations between protein expression patterns, tumor characteristics, and clinical outcomes within each molecular subtype. The results of the Kendall Tau correlation analysis are presented in Table 4 and Table 5.

In the TNBC group, a moderate positive correlation was observed between nuclear HSP70 and cytoplasmic MT (τ = 0.360). A weak negative correlation was found between nuclear HSP70 expression and tumor size (pT; τ = −0.250). Nuclear HSP70 expression was also negatively correlated with histological subtype (τ = −0.253). A weak positive correlation was observed between nuclear MT and cytoplasmic MT expression (τ = 0.231). Nuclear MT expression showed a moderate negative correlation with histological subtype (τ = −0.354). Additionally, weak negative correlations were observed between nuclear MT expression and metastatic location (τ = −0.137), and a moderate negative correlation between nuclear MT expression and mortality (τ = −0.311). Cytoplasmic MT expression showed moderate positive correlation with nuclear HSP70 expression (τ = 0.360). A strong negative correlation was observed between cytoplasmic MT expression and lymph node status (pN; τ = −0.506). Clinical outcome variables in TNBC demonstrated strong associations. Recurrence/Metastasis showed a very strong negative correlation with disease-free survival (DFS; τ = −0.749) and very strong positive correlations with metastatic location (τ = 0.854) and mortality (τ = 0.793).

In the Luminal A subgroup, nuclear HSP70 expression showed a moderate positive correlation with nuclear MT expression (τ = 0.341) and a weak positive correlation with cytoplasmic MT expression (τ = 0.251). A weak negative correlation was observed between nuclear HSP70 expression and mortality (τ = −0.148). Nuclear MT expression showed a moderate positive correlation with cytoplasmic MT expression (τ = 0.439). In addition, nuclear MT expression was positively correlated with tumor size (pT; τ = 0.275). Cytoplasmic MT expression showed weak positive correlations with tumor size (pT; τ = 0.219) and metastatic location (τ = 0.105). Regarding clinical outcomes, recurrence/metastasis showed a moderate negative correlation with disease-free survival (DFS; τ = −0.411). A very strong positive correlation was observed between recurrence/metastasis and metastatic location (τ = 0.980), as well as a strong positive correlation with mortality (τ = 0.622). Disease-free survival was negatively correlated with metastatic location (τ = −0.395) and mortality (τ = −0.380).

### 3.4. Analysis of Overall Survival (OS) and Disease-Free Survival (DFS) in Patients with Breast Cancer

#### 3.4.1. Kaplan–Meier Analysis of Overall Survival (OS)

Overall survival (OS) was analyzed using the Kaplan–Meier method to estimate the survival time course and compare patients with Luminal A and TNBC. OS was defined as the time from diagnosis to death or the end of follow-up. Patients alive at the end of follow-up were considered censored observations.

**Figure 2:**
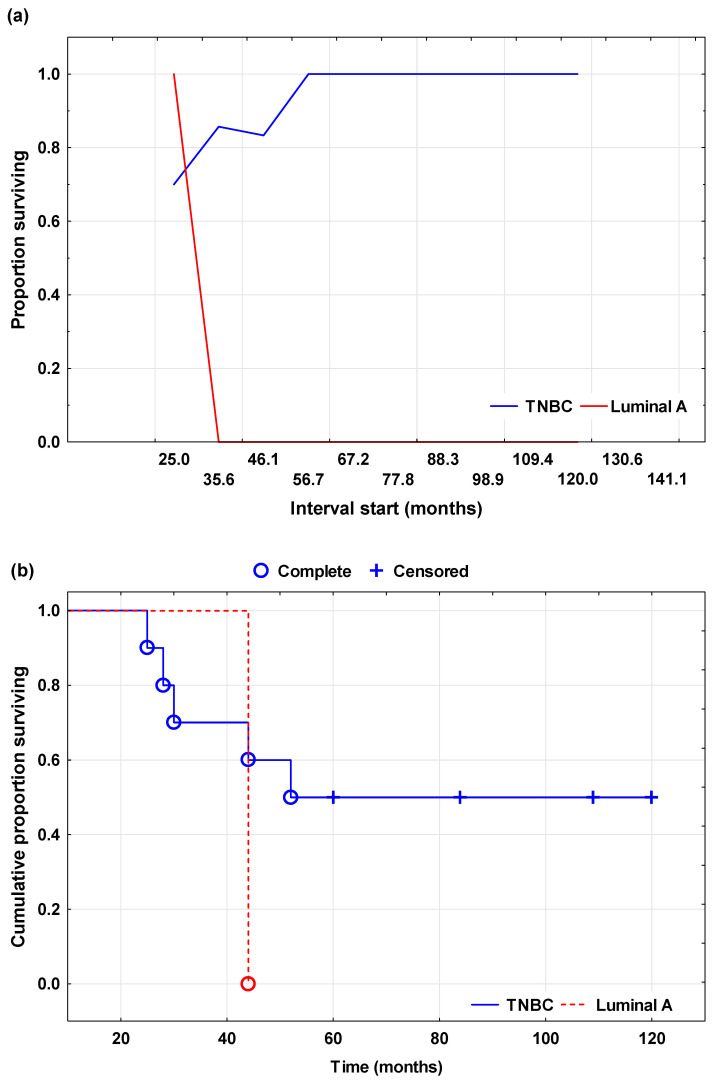
Kaplan–Meier analysis of overall survival (OS) in patients with Luminal A and triple-negative breast cancer (TNBC). (**a**) Survival rate by time interval in patients with Luminal A and TNBC, showing changes in the proportion of survivors during follow-up. (**b**) Kaplan–Meier overall survival curves, showing the cumulative probability of survival over time; circles indicate recorded events and “+” signs indicate censored cases.

The results of the Kaplan–Meier analysis are presented in Figure 2, which consists of two parts (Figure 2a,b). Figure 2a shows the survival proportion by time interval for both patient groups, providing insight into the distribution of recorded events during follow-up. Patients with Luminal A carcinoma maintained a higher survival proportion throughout most of the follow-up, while patients with TNBC showed a more pronounced decline in survival at earlier time intervals, indicating a less favorable early course of disease in this group. Figure 2b shows the cumulative Kaplan–Meier curve of OS, with recorded events and censored cases marked. Circles represent deaths, while “+” signs indicate censored patients. The curves clearly show the difference in survival patterns between groups, with TNBC patients experiencing a more pronounced decline in cumulative survival probability at earlier follow-up, while Luminal A patients showed a more gradual decline in survival, with a higher number of censored cases at later time points.

Statistical comparison of overall survival between groups was performed using the log-rank test. Although the Kaplan–Meier curves indicated a less favorable survival trend in the TNBC group, the difference did not reach statistical significance (log-rank test: χ^2^ = −0.66, *p* = 0.508). This result can be partially explained by the limited number of death events and differences in follow-up duration between groups.

#### 3.4.2. Kaplan–Meier Analysis of Disease-Free Survival (DFS)

Disease-free survival (DFS) was analyzed using the Kaplan–Meier method and defined as the time from diagnosis to the first recurrence or metastasis, or the end of the five-year follow-up period. Patients who did not experience recurrence or metastasis during follow-up were considered censored.

**Figure 3:**
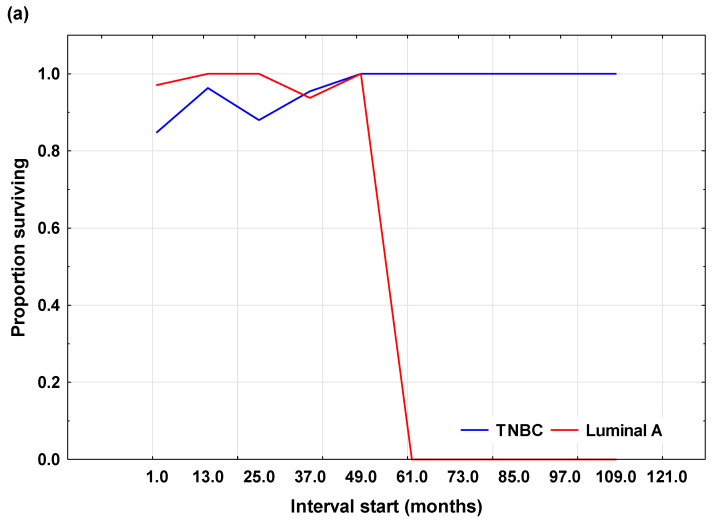

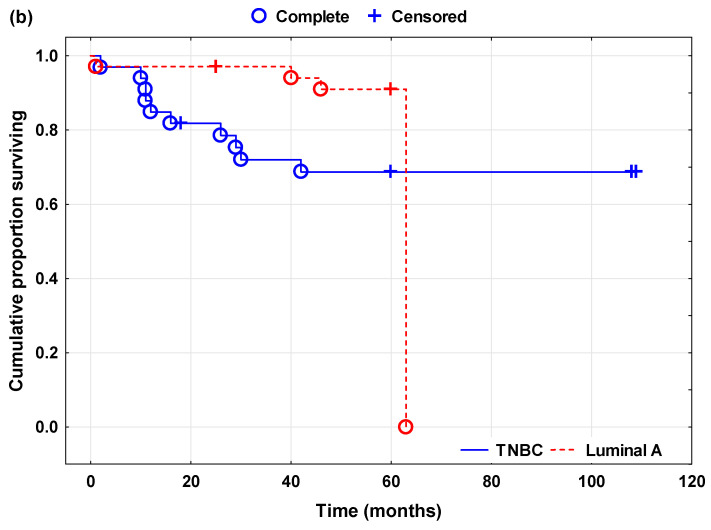
Kaplan–Meier analysis of disease-free survival (OS) in patients with Luminal A and triple-negative breast cancer (TNBC). (**a**) Disease-free survival rate over time in patients with Luminal A and TNBC, showing changes in the proportion of patients without recurrence or metastasis during follow-up. (**b**) Kaplan–Meier disease-free survival curves, showing the cumulative probability of remaining free of recurrence or metastasis over time; circles indicate recorded events and “+” signs indicate censored cases.

The Kaplan–Meier DFS analysis is shown in Figure 3, which includes two figures (Figure 3a,b). Figure 3a displays the proportion of patients without signs of disease over time for both groups. Patients with Luminal A carcinoma maintained a higher proportion of disease-free survival during follow-up compared to patients with TNBC, while patients with TNBC experienced earlier and more frequent disease progression. Figure 3b presents the cumulative Kaplan–Meier curves of DFS, with recorded events and censored cases indicated. Circles represent recurrence or metastasis, and “+” signs indicate censored patients. The curves illustrate differences in DFS patterns between groups, with the TNBC group showing a more pronounced decline in disease-free survival earlier in the follow-up period compared to the Luminal A group.

The log-rank test confirmed that the difference in DFS between the groups was not statistically significant (χ^2^ = 1.87; *p* = 0.062), although a clear trend toward shorter DFS in TNBC patients was observed. As with OS, the limited number of events and uneven follow-up duration among patients partly explain these results.

Overall, Kaplan–Meier analysis of both OS and DFS demonstrated distinct survival patterns between Luminal A and TNBC, characterized by earlier occurrence of events in the TNBC group. However, given the descriptive nature of Kaplan–Meier analysis and the limited statistical significance, further evaluation using multivariable Cox proportional hazards regression was performed to assess the independent prognostic significance of immunophenotype and other clinicopathological variables.

#### 3.4.3. Univariate Cox Regression Analysis of Risk Factors for Disease-Free Survival (DFS)

In a univariate Cox regression analysis, the individual influence of biological markers, clinicopathological characteristics and prognostic factors on the risk of recurrence or metastasis (DFS) in patients with TNBC and Luminal A BC was evaluated. The results are expressed as parameter estimate (B), standard error (SE), Chi-square value, *p*-value, hazard ratio (HR), and the corresponding 95% confidence interval (CI), and are presented in Table 6. An analysis of the influence of factors on OS was also performed, but due to the small number of events, the results were not included.

The biomarkers analyzed included nuclear HSP70 and cytoplasmic and nuclear metallothioneins (MTs). None of these markers showed a statistically significant effect on DFS. Specifically, nuclear HSP70 had an HR of 1.042 (95% CI 0.970–1.120; *p* = 0.255), cytoplasmic MT had a HR of 1.015 (95% CI 0.984–1.047; *p* = 0.350), and nuclear MT had an HR of 0.984 (95% CI 0.956–1.013; *p* = 0.269). These results suggest that, without considering other factors, HSP70 or MT protein levels do not significantly predict the risk of recurrence/metastasis in this sample. Age at diagnosis was also not a significant risk factor (HR 1.028; 95% CI 0.957–1.105; *p* = 0.451), indicating that patient age alone was not associated with an increased risk of recurrence or metastasis. Tumor histotype (ductal vs. other) also did not show a statistically significant impact on DFS (HR 0.623; 95% CI 0.171–2.264; *p* = 0.472), suggesting that histological type alone does not predict the risk of disease recurrence. Clinicopathological factors, including tumor size (pT) and lymph node status (pN), were not statistically significant predictors of DFS in univariate analysis. Tumor size greater than 5 cm (pT > 5) had an HR of 1.185 (95% CI 0.154–9.117; *p* = 0.871), while positive lymph node status (pN1–pN3) had an HR of 1.414 (95% CI 0.489–4.094; *p* = 0.523). In contrast, histological grade (G) was the only significant predictor of DFS. Higher grade tumors (G3) had a lower risk of recurrence/metastasis in this analysis (HR 0.168; 95% CI 0.052–0.548; *p* = 0.003), suggesting that tumor grade may be a strong predictor of disease behavior, even when analyzed individually. The Ki-67 proliferative index showed a trend toward a decreased risk for tumors with more than 20% positive cells (HR 0.336; 95% CI 0.111–1.014; *p* = 0.053), which is nearly statistically significant and may indicate a potential influence of cell proliferation on DFS. Finally, the immunophenotype of PHD (TNBC vs. Luminal A) showed a trend toward a reduced risk in TNBC (HR 0.347; 95% CI 0.108–1.115; *p* = 0.075), but the difference did not reach statistical significance.

#### 3.4.4. Multivariate Cox Regression Analysis of Risk Factors for Disease-Free Survival (DFS), Stratified by Histological Grade (G1–G3)

Multivariate Cox proportional hazards regression was performed to assess the independent effects of the proliferative index Ki-67 and PHD immunophenotype on the risk of recurrence and/or metastasis in BC patients, stratified by histological grade (G1–G3). Stratification by histological grade controlled for its effect on survival, while other variables were evaluated within each stratum.

The results of the multivariate analysis are presented in Table 7. For Ki-67, the hazard ratio (HR) was 0.557 (95% CI: 0.069–4.461, *p* = 0.58), indicating that a higher proliferative index did not have a statistically significant impact on the risk of recurrence/metastasis in this model. For the PHD immunophenotype, the HR was 1.234 (95% CI: 0.129–11.788, *p* = 0.855), also without statistical significance, indicating that the immunophenotype, when controlled for histological grade, was not an independent predictor.

The proportional hazards (PH) assumption was checked using Schoenfeld residuals. Testing showed that neither Ki-67 (*p* = 0.790) nor PHD (*p* = 0.085) significantly violated the PH assumption. Overall, the model showed a slight violation of the PH assumption (Overall *p* = 0.033), which can be expected due to stratification by histological grade and the relatively small sample size. Stratification ensures control of the effect of histological grade, while the proportionality of risk for other variables can be considered acceptable.

## 4. Discussion

### 4.1. The Role of Metallothionein and HSP70 in Carcinogenesis and Their Expression Patterns in TNBC and Luminal A Breast Cancer

This study analyzed the expression patterns of HSP70 and MTs in TNBC, Luminal A BC, and benign breast lesions to elucidate their biological roles and potential clinical relevance. The results demonstrated significantly increased nuclear expression of HSP70 in malignant breast tissues compared with fibroadenomas, while cytoplasmic MT expression was significantly reduced in both TNBC and Luminal A carcinomas. No significant differences in nuclear MT expression were observed between the analyzed groups. These findings suggest distinct and non-parallel roles of HSP70 and MTs in breast carcinogenesis and emphasize the importance of considering subcellular protein localization when interpreting their biological significance [23,29,42,43].

The elevated nuclear expression of HSP70 observed in TNBC and Luminal A carcinomas is consistent with its known role in cellular stress response mechanisms during malignant transformation. HSP70 participates in the stabilization of oncoproteins, inhibition of apoptosis, and stimulation of tumor cell survival under conditions of hypoxia, oxidative stress, and increased metabolic demand characteristic of the tumor microenvironment [23,35,44]. Previous immunohistochemical studies have consistently shown increased expression of HSP70 in BC compared to benign lesions, associating it with more aggressive tumor behavior, therapeutic resistance, and modulation of the immune response [44,45]. Moreover, chemotherapy-induced HSP70 secretion has been shown to promote protumorigenic macrophage polarization and immunosuppression, thereby facilitating tumor progression and treatment resistance [44,46]. In this context, the increased nuclear expression of HSP70 observed in malignant samples in the present study is consistent with its role in maintaining cell viability in a chronically stressful tumor environment. In the present study, no significant difference in nuclear HSP70 expression was found between TNBC and Luminal A subtypes, which suggests that elevated HSP70 expression primarily reflects malignant transformation rather than intrinsic molecular subtype-specific differences in BC [47]. This supports the concept of HSP70 as a general marker of cellular stress adaptation in malignant breast tissue rather than a reliable discriminator between biologically more aggressive and less aggressive subtypes.

Unlike HSP70, MT expression patterns showed a more complex and less linear relationship with malignancy. In this study, significantly reduced cytoplasmic MT expression was found in malignant breast tissues compared with fibroadenomas, while nuclear MT expression did not differ significantly between groups [42,48]. This finding deviates from reports describing increased overall MT expression in BC and its association with higher tumor grade, chemotherapy resistance, and worse outcomes [26,43]. This difference may be explained by the dual and context-dependent role of MTs in carcinogenesis, as well as differences in disease stage, molecular subtype, and subcellular localization [26,49]. MTs play a central role in metal ion homeostasis and protection against oxidative stress, exerting protective role in the early tumor development, while potentially promoting tumor cell survival and resistance to cytotoxic therapies in more advanced stages [26,48]. The reduced cytoplasmic expression of MTs observed in malignant tissues in this study may therefore reflect altered intracellular redistribution rather than overall decrease in MT levels. The preserved nuclear expression of MTs may indicate translocation to the nucleus, where they participate in the genomic protection and transcriptional regulation under permanent oxidative stress conditions [49,50]. Such redistribution has been proposed as one of the mechanisms of cellular adaptation in malignant cells and may explain inconsistent findings regarding the prognostic significance of MTs reported in the literature. Furthermore, the absence of significant differences in cytoplasmic or nuclear MT expression between TNBC and Luminal A carcinomas suggests that MT dysregulation represents general feature of malignant breast tissue rather than a subtype-specific phenomenon [26,48], consistent with previous studies highlighting the heterogeneity of MT expression in BC [43,49].

The significantly higher Ki-67 proliferation index observed in TNBC compared to Luminal A carcinomas further confirms the more aggressive biological profile of TNBC and aligns with extensive evidence linking elevated Ki-67 expression to increased proliferation and adverse clinicopathological features [40,51,52]. In the present cohort, although TNBC tumors showed both higher nuclear HSP70 expression and increased Ki-67 index values, these biological parameters did not demonstrate an independent prognostic effect in survival analyses. This further suggests their role in cellular adaptation rather than direct prediction of clinical outcome in TNBC, supporting their association with enhanced cellular stress and proliferative activity characteristic of this subtype [40,51].

### 4.2. Kaplan–Meier Survival Analysis (OS and DFS)

In this study, a Kaplan–Meier analysis of overall survival (OS) and disease-free survival (DFS) was performed to assess the potential association of HSP70 and MTs expression with treatment outcomes in BC patients. The analysis considered the subcellular localization of both proteins and the molecular subtypes of the tumor.

Kaplan–Meier curves showed a trend toward shorter OS and DFS in patients with TNBC compared to those patients with the Luminal A subtype, consistent with the well-known clinical course of these molecular subtypes [53,54]. Despite the observed differences in survival curves, the log-rank test did not show statistically significant differences in either OS or DFS between the analyzed groups, indicating a limited discriminatory capacity of this analysis within the studied cohort.

Analysis of survival in relation to HSP70 expression showed no significant association with either OS or DFS, regardless of the subcellular localization of the protein [55]. Although previous studies have reported an association between elevated HSP70 expression and poorer outcomes, especially in more aggressive BC subtypes, these findings have not been consistently confirmed in studies based on immunohistochemical assessment that take subcellular localization into account [22].

Similarly, Kaplan–Meier analysis demonstrated no statistically significant association between MTs expression and OS or DFS, irrespective of whether cytoplasmic or nuclear localization was considered [49]. Although some studies have described an association between increased MT expression and poorer prognosis or treatment resistance, especially in advanced disease, the available literature shows significant variability, likely reflecting differences in study design, methodological approaches and molecular subtype composition [26].

Taken together, the Kaplan–Meier survival analysis did not demonstrate a clear association between HSP70 and MTs expression and survival outcomes. These findings suggest that the expression of these proteins may be more closely related to adaptive cellular processes than to direct prognostic effects, supporting further evaluation using multivariate analytical approaches.

### 4.3. Cox Regression Survival Analysis (DFS)

To further evaluate the potential prognostic significance of the analyzed variables, a Cox regression analysis of disease-free survival (DFS) was performed. Before creating the multivariate model, a univariate Cox analysis was conducted, which included clinicopathological parameters as well as HSP70 and MTs expression with regard to their subcellular localization. Variables were included in the multivariate model based on the results of the univariate analysis and biological relevance.

In the univariate analysis, several clinicopathological parameters showed expected trends of association with DFS, while the expression of HSP70 and MTs did not show a statistically significant association with survival outcomes, regardless of their subcellular localization. These findings suggest a limited prognostic potential of the analyzed biomarkers in a univariate survival model, which is consistent with previous studies that failed to confirm an independent prognostic role of HSP70 or MTs [22,56].

In the multivariate Cox regression analysis, which simultaneously took into account multiple clinicopathological variables, the expression of HSP70 and MTs did not emerge as independent prognostic factors for DFS. In contrast, certain standard clinicopathological parameters retained prognostic relevance, which is consistent with current knowledge about their role in predicting disease outcome in BC [57,58].

The absence of an independent prognostic effect of HSP70 and MTs in the multivariate model can be considered in the context of their biological function as stress-responsive proteins involved in cellular adaptation mechanisms. While immunohistochemical assessment provides insight into their expression patterns, it may not fully capture dynamic functional changes relevant to disease progression [56,59].

Taken together, the results of univariate and multivariate Cox regression analysis suggest that HSP70 and MTs expression in BC does not provide independent prognostic information for DFS in this cohort. Nevertheless, their involvement in malignant transformation and cellular stress response supports further investigation within larger and methodologically standardized studies, integrating additional molecular and functional parameters, in line with the recent concepts of the integrative biomarkers development in oncology [60,61].

### 4.4. Biomolecular Mechanisms of HSP70 and Metallothionein Interaction in Breast Cancer-Biological Context

The combined activation of HSP70 and MTs may represent a complex adaptive network that enables tumor cells, especially in TNBC, to survive under oxidative, metabolic, and therapeutic stress. Both proteins play key roles in regulating cellular homeostasis, and their simultaneous activation has been increasingly described in aggressive tumor phenotypes and increased treatment-resistance behavior, primarily in experimental and preclinical models [32,62].

HSP70 acts as a molecular chaperone that prevents protein misfolding and aggregation under stressful conditions such as oxidative damage, hypoxia and exposure to cytotoxic agents [32,62]. In TNBC, HSP70 is frequently overexpressed in the cytoplasm and can also be detected on the tumor cell surface, making it a potential tumor-specific therapeutic target [63]. Increased expression of HSP70 has been associated with inhibition of apoptosis, enhanced metastatic potential, and reduced sensitivity to chemotherapy and radiotherapy [32,63,64]. In addition, HSP70 contributes to modulation of the tumor microenvironment by influencing both immune response, attracting both tumor-infiltrating lymphocytes and immunosuppressive populations such as myeloid-derived suppressor cells (MDSCs), thereby facilitating tumor progression [35,62]. Clinical studies have shown that elevated levels of HSP70 in immune cells within the tumor core correlate with higher tumor stage and grade, as well as increased expression of immunosuppressive markers such as PD-L1 and FOXP3+ regulatory T-cells in solid malignancies [35,62].

Metallothioneins are cysteine-rich proteins with a high affinity for divalent metal ions such as Zn^2+^ and Cu^2+^, enabling them to modulate intracellular metal availability and contribute to cellular defense mechanisms under conditions of oxidative and metabolic stress. In tumor cells, MTs interact with key transcriptional including MTF-1 and p53, thereby influencing gene regulation and expression, DNA repair, and apoptotic signaling pathways. Under stress conditions, MTs can translocate to the nucleus, where they participate in chromatin stabilization and transcriptional regulation, contributing to tumor cell survival [32].

The functional interplay between HSP70 and MTs represents an important mechanism of cellular adaptation in malignant conditions. HSP70 supports the stability and nuclear translocation of MTs under oxidative stress, while nuclear MTs regulate transcriptional programs related to proliferation, stress response, and DNA repair [32]. Both proteins are involved in the activation of redox-sensitive signaling pathways, especially NF-κB and Nrf2, which facilitate cellular adaptation to oxidative damage. Through stabilizations of NF-κB and regulation of zinc-dependent redox signaling, HSP70 and MTs promote the expression of genes associated with cell survival and antioxidant defense [32].

Although simultaneous activation of HSP70 and MT-related pathways has been linked to aggressive tumor characteristics and recurrence, particularly in TNBC, such observations are largely derived from preclinical and retrospective studies and show significant heterogeneity [32,64]. Therefore, their biological interaction should be viewed as part of a complex adaptive response of tumor cells, not as a direct indicator of clinical outcome.

Beyond these effects, HSP70 and MTs jointly contribute to the regulation of mitochondrial function and redox balance. HSP70 protects mitochondria from oxidative damage, while MTs participate in reactive oxygen species (ROS) scavenging, thus protecting mitochondrial DNA integrity and ATP production under metabolic stress [32,63]. In the Luminal A subtype of BC, which is characterized by less oxidative stress, activation of these adaptive mechanisms appears less pronounced, which is associated with more favorable biological features of this subtype [32,65]. The interaction of HSP70 and MTs may also influence the epithelial–mesenchymal transition (EMT) and the metastatic potential of tumors. HSP70 promotes the expression of EMT-related transcription factors such as Snail and Twist, while MTs modulate the activity of matrix metalloproteinases (MMPs), thereby facilitating extracellular matrix degradation and tumor cell invasion [32,65]. Targeting the HSP70–MT signaling axis in preclinical studies has shown potential in restoring sensitivity to apoptosis and reducing metastatic spread, although such approaches require caution due to the important physiological functions of these proteins in normal tissues [32,63,64]. In addition to their intracellular functions, HSP70 and MTs may also influence tumor progression through mechanisms of intercellular communication within the tumor microenvironment. Increasing evidence indicates that extracellular vesicles, particularly exosomes, play a key role in transmitting tumorigenic signals and modulating the immune response in the tumor microenvironment. Exosomes facilitate the transfer of bioactive molecules, including stress response proteins, nucleic acids, and lipids, between tumor cells, stromal cells, and immune cells, which may promote tumor invasiveness, immunosuppression, and metastatic potential [66,67,68,69]. HSP70 has been identified as a functional component of tumor exosomes, where it may regulate the immune response and induce protumorigenic signaling pathways in the tumor microenvironment [69,70]. Furthermore, exosome-mediated signaling has been implicated in regulating the metastatic spread of BC, and exosome-based strategies have shown potential in inhibiting postoperative metastasis in preclinical models [70,71,72]. Although exosomes were not directly analyzed in this study, the literature provides a possible mechanistic framework through which HSP70 and metallothioneins could influence the invasive and metastatic properties of TNBC via interactions in the tumor microenvironment, complementing their intracellular roles in the stress response.

Overall, these mechanisms do not imply a direct prognostic or predictive role of HSP70 and MTs within the scope of the present study, but rather provide a biological framework for interpreting their functional relevance across different molecular subtypes of BC. Further studies integrating functional, molecular, and clinical data are required to better elucidate these interactions.

## 5. Conclusions

This study contributes to the understanding of the role of stress-responsive proteins HSP70 and metallothionein in BC biology, with a special emphasis on the triple-negative subtype. Analysis of their expression and subcellular localization indicates the complexity of tumor cell adaptive mechanisms, which cannot be fully explained by simplified prognostic approaches based on single biomarkers.

Although HSP70 and metallothionein play important biological roles in cellular stress responses and malignant transformation, neither protein demonstrated independent prognostic value in the analyzed cohort. Differences in expression patterns across molecular subtypes reflect the heterogeneity of signaling networks involved in tumor adaptation to oxidative, metabolic, and therapeutic stress and appear to represent functional biological features rather than direct indicators of survival outcomes.

These findings should be interpreted in the context of certain limitations, including the retrospective study design, the relatively small cohort size, and the restriction of the study population to patients treated in Croatia, which may limit the generalizability of the results. Future studies integrating functional, molecular, and clinical data are needed to more precisely define the role of stress-responsive proteins in BC and support the development of more sophisticated biomarkers and therapeutic stratification strategies.

## Figures and Tables

**Table 1:** Demographic and clinicopathological characteristics of patients with triple-negative (TNBC), Luminal A breast cancer subtypes, and fibroadenomas (FA) as a control group (N = 90).

Characteristics of Carcinoma	TNBC ^a^ (*n* = 30)	Luminal A (*n* = 30)	FA ^b^ (*n* = 30)	Total
Age				
18–30	2	0	11	13
31–50	4	6	15	25
51–60	10	8	4	22
61–70	14	16	0	30
Average	61–70 (14 patients)	61–70 (20 patients)	20–40 (dominant)	–
Histological type				
Ductal	23	20	–	43
NST ^c^	0	10	–	10
Metaplastic	4	0	–	4
Medullary	3	0	–	3

^a^—triple-negative breast cancer; ^b^—fibroadenomas (control group); ^c^—invasive carcinoma of no special type.

**Table 2:** Clinical and histopathological characteristics, lymph node involvement, and Ki-67 proliferative index in triple-negative (TNBC) and Luminal A breast cancer subtypes (N = 60).

Characteristics of Carcinoma	TNBC ^a^ (*n* = 30)	Luminal A (*n* = 30)
Carcinoma size		
pT2 (2–5 cm)	28 (93%)	26 (87%)
pT3 (> 5 cm)	2 (7%)	4 (13%)
Lymph node status		
pN0 (negative)	20 (66%)	11 (37%)
pN1 (1–3 positive)	5 (17%)	11 (37%)
pN2 (4–6 positive)	2 (7%)	3 (10%)
pN3 (>7 positive)	2 (7%)	3 (10%)
pNx (unknown)	1 (3%)	2 (6%)
Histologic grade		
G1 ^b^	0 (0%)	18 (60%)
G2 ^c^	14 (47%)	8 (27%)
G3 ^d^	16 (53%)	4 (13%)
Ki-67 proliferative index		
>20% ^e^	25 (83%)	0 (0%)
<20% ^f^	5 (17%)	30 (100%)

^a^—triple-negative breast cancer; ^b^—well-differentiated carcinoma and closely resembles normal tissue; ^c^—moderately differentiated carcinoma, with some resemblance to normal tissue; ^d^—poorly differentiated carcinoma and significantly differs from normal tissue (“wild” or undifferentiated); ^e,f^—highly (rapid-growing) and low (slow-growing) proliferative carcinoma, respectively.

**Table 3:** Disease outcomes, including recurrence, metastasis, Disease-Free Survival (DFS), and Overall Survival (OS) in Triple-Negative (TNBC) and Luminal A breast cancer subtypes (N = 60).

Characteristics of Carcinoma	TNBC ^a^ (*n* = 30)	Luminal A (*n* = 30)
Recurrence/Metastases		
Yes	9 (30%)	4 (13%)
No	21 (70%)	26 (87%)
Metastasis location		
Combined	6	0
Bones	2	2
CNS	1	0
Liver	0	2
Lymph nodes	1	0
DFS ^b^		
Disease-free	19 (63%)	27 (90%)
Relapse of disease	11 (37%)	3 (10%)
OS ^c^		
Survivors	20 (67%)	29 (97%)
Deceased	10 (33%)	1 (3%)

^a^—triple-negative breast cancer; ^b^—disease-free survival (60-month survival); ^c^—overall survival.

**Table 4:** Kendall Tau analysis of the association between histological features, proliferation markers and clinical outcomes in triple-negative breast cancer (TNBC) subtype. Numbers highlighted in red in the table indicate statistically significant correlations with *p* < 0.05.

Variable	HSP70 (nuc.)	MT (nuc.)	MT (cyt.)	Age	pT	pN	G	Ki-67	Rec/Met	DFS	Met. Location	Mortality	Histotype
**HSP70 ^a^ (nuc.)**	1.000	0.163	0.360	0.019	−0.250	−0.144	−0.125	−0.046	0.194	−0.097	0.117	0.038	−0.253
**MT ^b^ (nuc.)**	0.163	1.000	0.231	−0.138	−0.153	−0.076	−0.033	−0.285	−0.135	0.173	−0.137	−0.311	−0.354
**MT ^c^ (cyt.)**	0.360	0.231	1.000	−0.357	−0.133	−0.506	−0.069	−0.075	0.072	−0.072	0.092	0.012	−0.089
**Age**	0.019	−0.138	−0.357	1.000	0.064	0.190	0.013	−0.009	−0.227	0.242	−0.229	−0.258	0.112
**pT ^d^**	−0.250	−0.153	−0.133	0.064	1.000	0.046	0.246	0.210	−0.167	−0.061	0.106	0.090	0.091
**pN ^e^**	−0.144	−0.076	−0.506	0.190	0.046	1.000	0.101	0.272	−0.150	0.098	−0.193	−0.035	0.027
**G ^f^**	−0.125	−0.033	−0.069	0.013	0.246	0.101	1.000	−0.171	0.112	−0.058	0.163	0.171	0.039
**Ki-67 ^g^**	−0.046	−0.285	−0.075	−0.009	0.210	0.272	−0.171	1.000	−0.140	0.108	−0.141	−0.167	0.215
**Rec/Met ^h^**	0.194	−0.135	0.072	−0.227	−0.167	−0.150	0.112	−0.140	1.000	−0.749	0.854	0.793	−0.099
**DFS ^i^**	−0.097	0.173	−0.072	0.242	−0.061	0.098	−0.058	0.108	−0.749	1.000	−0.750	−0.768	0.010
**Met. Location ^j^**	0.117	−0.137	0.092	−0.229	0.106	−0.193	0.163	−0.141	0.854	−0.750	1.000	0.763	−0.101
**Mortality**	0.038	−0.311	0.012	−0.258	0.090	−0.035	0.171	−0.167	0.793	−0.768	0.763	1.000	−0.013
**Histotype ^k^**	−0.253	−0.354	−0.089	0.112	0.091	0.027	0.039	0.215	−0.099	0.010	−0.101	−0.013	1.000

^a^—heat shock proteins in the nucleus; ^b^—metallothionein proteins in the nucleus; ^c^—metallothionein proteins in the cytoplasm; ^d^—carcinoma size; ^e^—lymph node status; ^f^—histologic grade; ^g^ —proliferative index; ^h^—recurrence/metastases; ^i^—disease-free survival; ^j^—metastasis location; ^k^—histological type.

**Table 5:** Kendall Tau analysis of the association between histological features, proliferation markers and clinical outcomes in Luminal A subtype. Numbers highlighted in red in the table indicate statistically significant correlations with *p* < 0.05.

Variable	HSP70 (nuc.)	MT (nuc.)	MT (cyt.)	Age	pT	pN	G	Ki-67	Rec/Met	DFS	Met. Location	Mortality	Histotype
**HSP70 ^a^(nuc.)**	1.000	0.341	0.251	0.003	−0.132	0.034	0.186	*–*	0.057	0.205	0.056	−0.148	0.024
**MT ^b^ (nuc.)**	0.341	1.000	0.439	−0.022	0.275	0.128	0.177	*–*	−0.119	0.048	−0.103	−0.175	0.015
**MT ^c^ (cyt.)**	0.251	0.439	1.000	0.074	0.219	−0.043	0.018	*–*	0.099	0.007	0.105	−0.026	0.098
**Age**	0.003	−0.022	0.074	1.000	0.105	0.121	−0.046	*–*	−0.035	−0.123	−0.044	−0.146	−0.064
**pT ^d^**	−0.132	0.275	0.219	0.105	1.000	0.389	−0.031	*–*	0.150	−0.147	0.171	0.106	−0.079
**pN ^e^**	0.034	0.128	−0.043	0.121	0.389	1.000	0.028	*–*	0.293	−0.068	0.305	0.179	−0.099
**G ^f^**	0.186	0.177	0.018	−0.046	−0.031	0.028	1.000	*–*	−0.204	0.027	−0.200	−0.186	−0.028
**Ki-67 ^g^**	*–*	*–*	*–*	*–*	*–*	*–*	*–*	1.000					
**Rec/Met ^h^**	0.057	−0.119	0.099	−0.035	0.150	0.293	−0.204	*–*	1.000	−0.411	0.980	0.622	−0.079
**DFS ^i^**	0.205	0.048	0.007	−0.123	−0.147	−0.068	0.027	*–*	−0.411	1.000	−0.395	−0.380	0.020
**Met. Location ^j^**	0.056	−0.103	0.105	−0.044	0.171	0.305	−0.200	*–*	0.980	−0.395	1.000	0.624	−0.061
**Mortality**	−0.148	−0.175	−0.026	−0.146	0.106	0.179	−0.186	*–*	0.622	−0.380	0.624	1.000	−0.133
**Histotype ^k^**	0.024	0.015	0.098	−0.064	−0.079	−0.099	−0.028	*–*	−0.079	0.020	−0.061	−0.133	1.000

^a^—heat shock proteins in the nucleus; ^b^—metallothionein proteins in the nucleus; ^c^—metallothionein proteins in the cytoplasm; ^d^—carcinoma size; ^e^—lymph node status; ^f^—histologic grade; ^g^—proliferative index; ^h^—recurrence/metastases; ^i^—disease-free survival; ^j^—metastasis location; ^k^—histological type.

**Table 6:** Univariate Cox proportional hazard analysis of biomarkers (HSP70, MT, Ki-67), clinicopathological characteristics (age, histotype, carcinoma stage), and prognostic parameters (recurrence/metastasis, PHD immunophenotype) on disease-free survival (DFS) in patients with triple-negative (TNBC) and Luminal A breast carcinoma. Results include parameter estimate (B), standard errors (SE), hazard ratios (HR) with 95% confidence intervals, and *p*-values. Numbers highlighted in red in the table indicate statistically significant correlations with *p* < 0.05.

Variable	Coding/Levels	Parameter Estimate (B)	Standard Error (SE)	Chi-Square	*p*-Value	Hazard Ratio (HR)	95% CL HR (Lower–Upper)
HSP70 ^a^ (nuc.)	continuous	0.042	0.037	1.295	0.255	1.042	0.970–1.120
MT ^b^ (cyt.)	continuous	0.015	0.016	0.873	0.350	1.015	0.984–1.047
MT ^c^ (nuc.)	continuous	−0.016	0.015	1.221	0.269	0.984	0.956–1.013
Age at diagnosis	continuous	0.028	0.037	0.569	0.451	1.028	0.957–1.105
Histotype	0 = ductal; 1 = others	−0.473	0.658	0.516	0.472	0.623	0.171–2.264
pT ^d^	0 = pT 2–5; 1 = pT >5	0.085	0.521	0.027	0.871	1.185	0.154–9.117
pN ^e^	0 = pN0; 1 = pN1–3	0.173	0.271	0.408	0.523	1.414	0.489–4.094
G ^f^	0 = G1–2; 1 = G3	−0.892	0.301	8.751	0.003	0.168	0.052–0.548
Ki-67 ^g^	0 = <20%; 1 = >20%	−0.545	0.282	3.748	0.053	0.336	0.111–1.014
PHD (immunophenotype)	0 = Luminal A; 1 = TNBC	−0.529	0.297	3.160	0.075	0.347	0.108–1.115

^a^—heat shock proteins in the nucleus; ^b^—metallothionein proteins in the cytoplasm; ^c^—metallothionein proteins in the nucleus; ^d^—carcinoma size; ^e^—lymph node status; ^f^—histologic grade; ^g^—proliferative index.

**Table 7:** Multivariate Cox proportional hazards analysis of Ki-67 and PHD immunophenotype on disease-free survival (DFS), stratified by histological grade (G1–G3) in patients with triple-negative (TNBC) and Luminal A breast carcinoma. Results include parameter estimate (B), standard error (SE), Wald chi-square with degrees of freedom (df), hazard ratios (HR) with 95% confidence intervals, and *p*-values.

Variable	Coding/Levels	Parameter Estimate (B)	Standard Error (SE)	Chi-Square	*p*-Value	Hazard Ratio (HR)	95% CL HR (Lower–Upper)
Ki-67 ^a^	0 = <20%; 1 = >20%	−0.293	0.531	0.304	0.581	0.557	0.069–4.461
PHD (immunophenotype)	0 = Luminal A; 1 = TNBC	0.105	0.576	0.033	0.855	1.234	0.129–11.778

^a^—proliferative index.

## Data Availability

The data supporting the findings of this study are available from the corresponding author upon reasonable request.

## Abbreviations

The following abbreviations are used in this manuscript:

Abbreviation	Full Name
A20	Tumor Necrosis Factor Alpha-Induced Protein 3
BC	Breast Cancer
CI	Confidence Interval
CNS	Central Nervous System
CSC	Cancer Stem Cells
DAPI	4′,6-Diamidino-2-Phenylindole
DFS	Disease-Free Survival
EMT	Epithelial–Mesenchymal Transition
ER	Estrogen Receptor
FA	Fibroadenoma
FFPE	Formalin-Fixed Paraffin-Embedded
FOXP3+	Forkhead Box P3–Positive
HER2	Human Epidermal Growth Factor Receptor 2
HSP70	Heat Shock Protein 70
HR	Hazard Ratio
IgG1	Immunoglobulin G1
IHC	Immunohistochemistry
Ki-67	Ki-67 Proliferation Index
MDSC	Myeloid-Derived Suppressor Cells
MMP	Matrix Metalloproteinase
MT	Metallothionein
NF-κB	Nuclear Factor Kappa-Light-Chain-Enhancer of Activated B Cells
NST	No Special Type
Nrf2	Nuclear Factor Erythroid 2–Related Factor 2
OS	Overall Survival
PD-L1	Programmed Death-Ligand 1
PH	Proportional Hazards
PR	Progesterone Receptor
rhHSP70	Recombinant Human Heat Shock Protein 70
ROS	Reactive Oxygen Species
SE	Standard Error
TMA	Tissue Microarray
TNBC	Triple-Negative Breast Cancer
TNM	Tumor–Node–Metastasis
